# Tandem reef restoration using corals and sea urchins: Building complex habitat for herbivores

**DOI:** 10.1371/journal.pone.0325468

**Published:** 2025-06-10

**Authors:** Catherine Lachnit, Emily Esplandiu, Joshua Patterson, Diego Lirman

**Affiliations:** 1 Department of Marine Biology and Ecology, Rosenstiel School of Marine, Atmospheric, and Earth Science, University of Miami, Key Biscayne, Florida, United States of America; 2 Fisheries and Aquatic Sciences, School of Forest, Fisheries, and Geomatics Sciences, University of Florida/IFAS, Gainesville, Florida, United States of America; King Abdulaziz University, SAUDI ARABIA

## Abstract

Amidst the decline of coral reef ecosystems, restoration practitioners are expanding their focus to incorporate key reef community components, such as grazers, to improve site conditions and the long-term survivorship of restored corals. We investigated the use of hatchery-propagated *Diadema antillarum* as well as two other locally abundant urchin species, *Lytechinus variegatus* and *Echinometra viridis*, for coral-urchin tandem reef restoration in Florida, USA. Urchins were deployed onto reef plots at various stages of *Acropora cervicornis* restoration and provided artificial cement refuges to evaluate retention and herbivory rates. Retention of urchins was low and variable among species. After 42 days, retention was 22% for *E. viridis*, 7% for *D. antillarum*, and 0% for *L. variegatus*. Retention was influenced by plot complexity (restoration state) and was significantly higher in high-complexity plots for *D. antillarum* and *E. viridis*. Within plots, refuge types did not have a significant influence on urchin retention. A reduction in macroalgal cover was only observed on plots with relocated *E. viridis* when densities were maintained > 0.4 urchins m^-2^. A second deployment of *D. antillarum,* with urchins caged for a month prior to release, resulted in significantly higher urchin retention. Within cages, grazing and the consumption of coral tissue were influenced by urchin density. At low urchin densities (4 urchins m^-2^) macroalgae cover remained high and corals were overgrown by algae. At intermediate densities (12 urchins m^-2^) algae were reduced and the growth of corals was maximized. At the highest densities (40 urchins m^-2^), algal cover was reduced but urchins caused tissue mortality as a result of over-grazing, highlighting the importance of maintaining relocated urchins at adequate densities to maximize the benefits of tandem restoration. Thus, if retention can be improved and urchins maintained at intermediate densities, the tandem restoration of corals and sea urchins could increase the efficacy of reef restoration.

## Introduction

After over two decades of coral propagation and reef restoration in Florida, USA, and the Caribbean, advances in husbandry, genotype selection, and outplanting methods have resulted in high performance of restored corals, with *Acropora cervicornis* survivorship commonly exceeding 80% during the first 1–2 years post-outplanting [1–3]. However, initial survivorship generally declines, sometimes rapidly, beyond the first two years, indicating that current reef conditions still pose challenges for long-term coral survivorship [4]. Once outplanted, corals become an integral component of the reef community and are exposed to the same stressors that influence the growth and mortality of wild corals. In cases where the sources of coral decline include large-scale drivers (e.g., disease, temperature anomalies, and storms), restoration practitioners have novel options like genotype selection, selective breeding, assisted relocation and migration, and stress hardening available to improve the viability of restored corals [5]. These approaches can maximize coral survivorship by placing species, genotypes, and phenotypes in the “right” environments [6].

Restoration practitioners also have the option of optimizing coral survivorship by improving the local conditions at the selected restoration locations [7,8]. A study by van Woesik et al. [3], showed that the survivorship of transplanted *A. cervicornis* was lower when in contact with macroalgae (mainly *Dicyota*). Not only do macroalgae compete with the growth and survivorship of adult coral colonies, but they can also inhibit coral recruitment and settlement, an essential process for the resilience and recovery of coral reef ecosystems [9]. These observations, along with a wealth of historical studies, have shown that macroalgal competition can lead directly and indirectly to coral mortality [10–12]. This has prompted restoration practitioners to reevaluate and expand current reef restoration practices [13,14]. One approach being considered is the tandem restoration of corals and macrograzers (e.g., sea urchins, crabs) to improve coral survivorship and growth, while restoring coral cover and key ecological processes like herbivory [15–20]. This is especially important in regions like Florida, USA, where coral cover has declined to historical low values and macroalgae often occupy > 50% of the benthos [21].

One of the key early drivers of coral declines and subsequent ecological phase-shifts on Caribbean reefs was the mass mortality of the keystone herbivore *Diadema antillarum* during 1983–1984, thought to be the most extensive and severe die-off recorded for a marine invertebrate [22]. This event led to a drastic reduction in *D. antillarum* populations by 95–100%, closely followed by a surge in macroalgal cover across the Caribbean, ranging from 100–250% [22–24]. Coral-dominated reefs require high levels of herbivory (and low nutrient levels) to prevent macroalgal overgrowth and competition [25]. Once a coral reef transitions to a fleshy, macroalgal-dominated state, there is a loss in resilience and reef complexity, making it difficult to recover lost ecosystem functions [26,27]. In the Caribbean there has been a shift from low-canopy algae (such as algal turfs and biofilms) to more competitive fleshy macroalgae, which continue to correlate with a loss in coral cover [28]. Taxa like *Dictyota* and *Lobophora,* previously controlled by *D. antillarum* grazing, have proliferated on reefs, especially in areas where fish grazers have been removed by overfishing [29–31]. Macroalgae can compete with coral colonies and coral larvae for space, inhibiting coral settlement, growth, and survivorship, likely making the initial phase shift persistent over time and leading to a state of “reef flattening” [10,11,32,33]. *D. antillarum* populations in Florida and throughout most of the Caribbean have not recovered after the initial mass mortality event and remain at diminished densities of 1–7% of pre-mortality prevalence [24,34–37].

Active interventions to restore *D. antillarum* populations have thus been identified as a crucial reef restoration need, as it could take decades for urchin recovery to occur naturally [17,38]. The lack of recovery is primarily linked to persistent failures in fertilization and recruitment driven by an absence of source populations and compounded by inadequate settlement and habitat availability [39–41]. This was further reinforced, as some recovering *D. antillarum* populations were affected by another mortality event in 2022 [42,43].

Previous efforts to restock urchins have achieved differing levels of success. These efforts included translocating wild *D. antillarum* [18,44–47], collecting wild settlers and rearing them until restocking size [48–50], and spawning captive individuals and rearing them from gametes [51–53]. Present populations of *D. antillarum* are insufficient to support large-scale translocations. One alternative for restocking depleted populations is using hatchery-propagated individuals [47,54]. While the use of hatchery-raised *D. antillarum* was initially questioned [55] and spawning/settlement success was limited [56], researchers have continued to explore the potential for using reared urchins for restoration [51,52,57,58]. This study is the first to report on the efficacy and retention of hatchery-propagated *D. antillarum* within coral restoration plots and employs the largest hatchery-propagated individuals stocked to date. Using hatchery-propagated *D. antillarum* allows for restocking without affecting wild populations. Ultimately, if *D. antillarum* restoration proves inefficient in the long term and at meaningful scales, utilizing other echinoid species that have similar ecological functions may become an important restoration option [48,59].

In Florida, *Lytechinus variegatus* and *Echinometra viridis* are two echinoid species that have similar ecological functions as *D. antillarum* [59]. Both species have stable wild populations, thus allowing for direct translocation. Translocation of these species may even be more effective as they are typically less mobile than *D. antillarum* [60,61], potentially leading to higher site retention. *L. variegatus* is effective at controlling macroalgal overgrowth and enhancing coral survivorship and growth in lab conditions, but their grazing ability in natural reef environments has not yet been evaluated [62]. In Panama, *E. viridis* have filled the functional role of *D. antillarum* and were found to be effective at limiting macroalgal growth [63]. While clearly a positive finding, these herbivores were found in high quantities in the Bocas del Toro reefs that were dominated by the coral *Agaricia* which provides suitable habitat for small-bodied urchins and are different from low-cover reefs in Florida and elsewhere in the Caribbean. Although neither species, *L. variegatus* or *E. viridis*, have naturally filled the functional role left by *D. antillarum* within Florida reefs, it is worth investigating if, with assisted translocation, they can fulfill this empty niche at relevant restoration scales.

Two of the biggest hurdles that echinoid restocking has faced are low survival and site retention. *D. antillarum* are known to seek shelter during the day in highly complex areas to avoid predation and leave their refuge to graze at night [64]. Initial *D. antillarum* restoration efforts recorded complete disappearance of translocated urchins within 24 hours – 1 week, or high rates of predation or emigration over the span of a few months, largely attributed to low reef complexity [46,47,65]. Methods to enhance shelter availability have included the use of artificial structures [18,50], translocating urchins within coral restoration sites [44,66], and the use of cages to prevent emigration and urchin predation by fish [48,49,66]. However, these methods have had limited and variable success, creating the need to explore additional methods to maximize urchin retention on restored plots. Here, restoration plots representing early- and late-stage *Acropora cervicornis* restoration states, along with habitat-enhancing structures with varying structural complexity were used to evaluate the role of physical structure on echinoid retention and grazing patterns, to inform the most efficient timing for urchin deployment within restored plots. Moreover, the use of temporary cages was evaluated as a tool to enhance long-term urchin retention. This is the first study to evaluate the retention rates of three Caribbean echinoid species (*Diadema antillarum*, *Lytechinus variegatus*, and *Echinometra viridis*) deployed into restoration plots and their potential benefits on macroalgal biomass and coral growth and survivorship. Additionally, the role of restoration state (initial and advanced) and refuge types (live coral, dead coral, cement structures) were explored to provide needed guidance for restoration practitioners on best practices to maximize the success of coral-urchin tandem restoration.

## Materials and methods

### Site description

All experiments were conducted at a single reef site in Miami Beach, Florida, USA (25.833° N, 80.107° W, depth = 7 m). This site is characterized by its low topographical complexity (< 20 cm average height/rugosity across the site) that provides a uniform habitat where the effects of introduced structure on urchin retention would be maximized. The site has persistent, high macroalgal cover (~ 60%) and low coral cover (< 5%), so that the impacts of urchin grazing can be easily detected if present. Common macroalgae at the site include *Dictyota, Halimeda, Caulerpa,* and mixed-species algal turfs. This site has been used for *Acropora* restoration in the past with high success (unp. results). The availability of urchins for this experiment was limited so the number of deployments that could be completed was prioritized over site replication. Adding other sites would make the results more widely applicable but would have reduced the power of the experiments conducted.

The Miami Beach site was used to test retention rates and herbivory effects of three urchin species, *D. antillarum*, *L. variegatus*, and *E. viridis.* During preliminary site surveys, only one large *D. antillarum* and no individuals of the other two urchin species were encountered. In this study, only one urchin species was deployed at a time, and all urchins remaining at the end of each experiment were relocated to another reef to avoid multi-species interactions. The plots and algal community were allowed to recover for at least 30 days between deployments. The sequential deployment of the different urchin species introduced a seasonality effect on macroalgal and grazing dynamics. However, comparisons of algal communities and grazing patterns were only analyzed and discussed here within deployments and were not compared among deployments, removing the influence of seasonality on these metrics. However, this does not discount the possibility that there may be seasonality influences on urchin retention patterns not accounted for in this study. This potential factor could be addressed when additional urchins and restored corals become available so that all experiments can be completed at the same time. The number of urchins deployed per plot and treatment type varied by species based on urchin availability. All research was conducted under and complied with a Florida Fish and Wildlife Conservation Commission Special Activity License (SAL-22-1794-SCRP).

### Urchin retention and grazing

#### Species comparisons.

To test how natural and artificial structures with differing structural complexity influence echinoid retention and grazing, three site plot types (low complexity, high complexity, and control), with two replicates each, were installed at the Miami Beach reef. Each experimental site plot consisted of a 3 m x 5 m grid, with structures (corals and cement structures) deployed at 1-m spacing, for a total of 15 structures per plot. This spacing was chosen since *D. antillarum* that exhibit homing behaviors often stay within a foraging range of approximately 1 m^2^ [64]. Site plots were separated by at least 10 m to limit echinoid movement among neighboring plots. The habitat-enhancing structures included live *A. cervicornis*, dead *A. cervicornis* skeletons, and concrete domes. The three treatments were placed throughout each site plot in a Latin square design to randomize placement, with each structure treatment having five replicates within each site plot.

**Fig 1:**
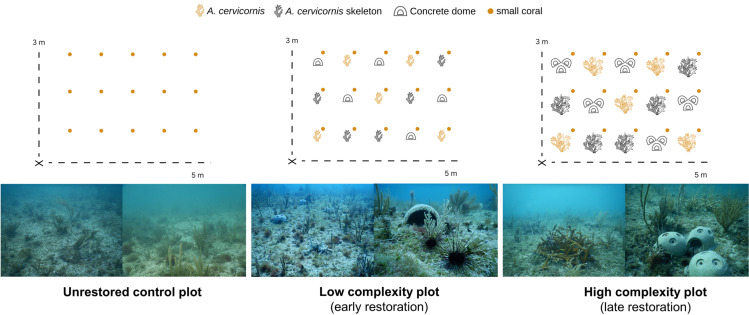
Experimental plots at the Miami Beach reef site. Plots were 3 x 5 meters. In the two experimental plots (low complexity (*n *= 2) and high complexity (*n *= 2)) there were three different structure treatment types: live *Acropora cervicornis*, dead *A. cervicornis* skeleton, and an artificial concrete dome (*n *= 5). A *Porites porites* coral fragment (1 cm^2^) was placed adjacent to each habitat structure. The unrestored control plots (*n *= 2) did not receive any added structure but still received small coral fragments at the 1-meter mark.

The low-complexity plots received five replicates of each structure treatment (i.e., five colonies of living *A. cervicornis*, five dead *A. cervicornis* skeletons, five single concrete domes; Fig 1). Live and dead *A. cervicornis* in low-complexity plots had an average linear length and width of ~15 and 10 cm respectively. The concrete domes, designed and manufactured by Reef Cells, measured 14 x 16 cm, with three 7 x 8 cm openings evenly placed around the circumference of the structure. The high-complexity plots also received five replicates of each structure treatment (Fig 1). The live *A. cervicornis* treatments consisted of clusters of 5–10 larger coral colonies, deployed in contact with each other to create a footprint of approximately 0.5 m^2^. The dead *Acropora* treatment was composed of clusters of dead *A. cervicornis* skeletons with a similar footprint (0.5 m^2^). The artificial structures in the high-complexity plots were deployed as three dome units placed together in a triangle configuration (Fig 1). The low-complexity plots mimic a state of early or initial restoration, with approximately 1.5% coral cover, whereas the high-complexity plots are representative of a more developed restoration state, 2–3 years after initial outplanting, with ~ 25% coral cover (Fig 1). Control site plots were established to compare natural changes in macroalgal biomass and coral condition to changes caused by echinoid deployment and herbivory. These control plots lacked added structures or *A. cervicornis*. To assess whether the presence and grazing of urchins influenced the survivorship and growth of small corals, 15 fragments of *Porites porites* (1–3 cm in diameter) were added using cement within each plot (Fig 1).

The *D. antillarum* in this experiment were reared from gametes at The Florida Aquarium [51,52] using broodstock collected from the Florida Keys. The *D. antillarum* had an average test diameter (TD) of 24.2 ± 3.1 mm and were deployed into the site plots in September 2022. Seventy five *D. antillarum* were placed in the high-complexity plots and 50 were placed in the low-complexity plots. The *L. variegatus* had an average TD of 62.1 ± 7.1 mm, were collected from seagrass beds within Biscayne Bay, and deployed to Miami Beach in November 2022. Fifty five *L. variegatus* were deployed into the high-complexity plots and 35 urchins were placed into the low-complexity plots. The *E. viridis* were collected from reef sites within 100 km and were deployed in March 2023. *E. viridis* were deployed with an average TD of 27.9 ± 4.2 mm. Thirty urchins were distributed into the high-complexity plots and 30 in low-complexity plots. Limited urchin availability resulted in different numbers of urchins being deployed across treatments to maximize data collection; these differences we accounted for statistically.

At the time of deployment, divers placed urchins adjacent to the structures by hand. The control plots did not receive any urchins during these experiments and served to monitor benthic macroalgal composition and coral growth in the absence of urchins.

#### Caging and site acclimation.

**Fig 2:**
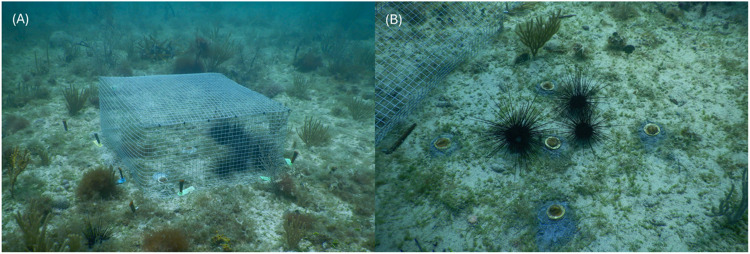
Example photographs of the urchin cages. **(A)** Galvanized mesh cages (0.25 m^2^) nailed in place at the time of urchin deployment **(B)** *Diploria labyrinthiformis* coral fragments and *Diadema antillarum* at the time of deployment before the cage was nailed in place.

Previous studies suggest that keeping urchins within cages after deployment to a new site can enhance retention after the barriers are removed caused by induced homing behavior [48,49]. To test whether this approach led to higher retention at the Miami Beach site and evaluate the effects of urchin density on algal cover, a second *D. antillarum* deployment that included caged and uncaged urchins was completed. Cages (50 cm x 50 cm x 11 cm; 1/2 inch openings; Fig 2A) were built using galvanized metal mesh. The bottom of the cage was open to allow urchins to graze the benthos. Cages were nailed in place at the time of each urchin deployment. Five cages received one urchin, five cages received three urchins, and four cages received 10 urchins, corresponding to densities of 4 urchins m^-2^, 12 urchins m^-2^, and 40 urchins m^-2^, respectively. Within each cage, five *Diploria labyrinthiformis* corals (average size = 3.4 cm^2^) were cemented to the reef to document impacts of urchin grazing on coral survivorship and growth (Fig 2B). Uncaged control plots (*n* = 2), with 10 *D. labyrinthiformis* per plot, were used to assess and evaluate algal cover and coral growth in the absence of urchin grazing.

In August 2023, 120 cultured *D. antillarum* with an average TD of 51.0 ± 8.9 mm were released at the Miami Beach reef. Half of the urchins (*n* = 60) were placed into the high, low, and control plots previously described, and the other half (*n* = 60) were placed into the 14 cages, as described above. The urchins that were deployed into the restoration plots were monitored until they all emigrated, after which the caged urchins were released and transferred into the same plots. The retention of urchins that were previously caged was compared to the retention of urchins that were directly deployed into the restoration plots.

### Monitoring

Visual surveys of the restored *Acropora cervicornis* at the site showed that no coral mortality was observed during this experiment. Urchins were surveyed 24 hours after release, and subsequently at 7, 14, and 42 days (*L. variegatus* were not surveyed at 24 hours, and *E. viridis* were not monitored at day 7 due to bad weather). The number of urchins and structure type in which they were found were recorded by divers within each plot. In addition to the area within each site plot (15 m^2^), an expanded area (10 m in radius) around each plot was surveyed to identify urchins that may have emigrated from the plot. The habitat outside of the site plots was mostly flat hardbottom with algal mats and sparse cover of soft corals, similar to the control plots. To quantify algal cover, 15 benthic photos were tracked per plot by placing a 0.25 m^2^ quadrat around each structure or at the center stake in control plots. The images were collected prior to urchin deployment and again during each subsequent survey. Benthic images were analyzed using the software Coral Point Count with Excel extensions (CPCe), with 25 random points overlaid within each image to calculate macroalgal percent cover [67]. The diameter (cm) of the small *Porites porites* were assessed using scaled photographs at deployment and after 42 days using ImageJ [68].

In the caging experiment, images of the benthos inside the cages were collected prior to deployment and 28-days after urchin deployment. The diameter (cm) of the small *D. labyrinthiformis* deployed in these experiments was assessed using scaled photographs taken at deployment and after 28 days using ImageJ [68].

### Statistical analyses

The experimental units in this study are the individual treatment types (*A. cervicornis*, dead *A. cervicornis* skeletons, concrete domes; *n* = 5 per treatment) placed within high- and low-complexity plots (*n* = 2 plots per complexity type) and data were combined within plot type. The low plot replication limits the statistical power of our analyses but is a direct consequence of low urchin availability. To account for variation in initial urchin numbers, retention was modeled using proportional data (i.e., the number of urchins retained relative to the initial number deployed) [69,70].

A generalized linear model (GLM) with a binomial distribution was used to compare the proportion of urchins retained among structure types and complexity plots over time. Survey timepoint, site complexity plot, and structure type were used as fixed categorical predictors. Models were tested with random effects for site plot and time to account for repeated measurements, but low variance and drop in deviance tests, indicated that they did not add to the models. A separate GLM was built for each urchin species. Models were fit using the ‘*glm*’ function within the *lme4* package. When complete or quasi-complete separation was detected in the models, GLMs were fit using the ‘*brglm*’ package.

A binomial generalized mixed effects linear model (GLMM) was built for each urchin species to compare changes in the percent macroalgal cover among treatments over time, with structure type and survey timepoint as fixed categorical predictors, initial macroalgal cover as a covariate, and site plot and survey timepoint as a random effect. Percent cover data were grouped by treatment plot (with and without urchins) for the 14 and 42-day time points. Models were fit using the ‘*glmer*’ function within the *lme4* package. For each model, significance was evaluated via likelihood ratio testing and additional post hoc analysis was conducted to compare treatments and individual time points using the ‘*emmeans*’ function.

The effect of urchin grazing on coral growth (final coral diameter – initial coral diameter, cm) was evaluated using a linear mixed model with cage as a random effect. A linear model was conducted to compare urchin density influence on the relative reduction of macroalgae within cages. When normality assumptions were not met, a non-parametric Kruskal-Wallis test was performed, followed by a Dunn’s test with Bonferroni correction for multiple comparisons. All statistical tests were performed using R software version 4.3.0. and significance was established at p* *< 0.05.

## Results

### Urchin retention and grazing

#### Species comparisons.

**Fig 3:**
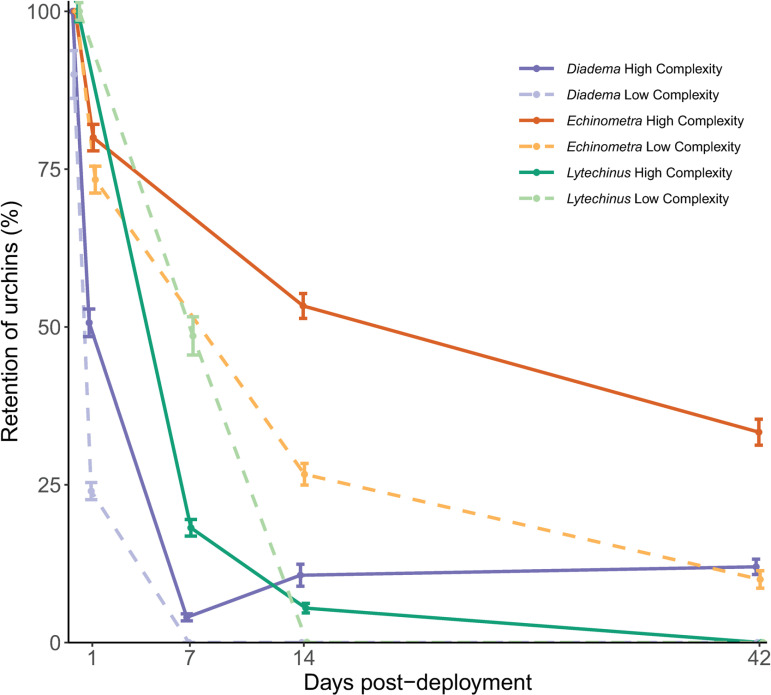
Retention of urchins. Percentage of urchins (±SE) that remained within the site plots over time for all three species. Each color represents a different species (*Diadema antillarum, Echinometra viridis, Lytechinus variegatus*). Solid and dashed bars represent differing plot complexities (high and low).

Retention of *D. antillarum* was higher in the high-complexity plots compared to the low-complexity plots throughout the experiment (Fig 3, GLM, p < 0.05). One day after deployment, *D. antillarum* had 50% retention in the high-complexity plots compared to 24% retention in the low-complexity plots, for an overall retention of 40%. During these surveys an additional 18% of the urchins were found outside of the plots. By day 42, there was only 7% retention, with all remaining urchins found exclusively within the high-complexity plots. There was no difference in microhabitat selection/retention among the different structure types (live coral, coral skeleton, concrete domes; GLM, p > 0.05). Retention declined over time and retention at day 1 was higher compared to all other monitoring time points (day 7, day 14, and day 42, post hoc analysis p* *< 0.05), while all the other survey time points were not different from one another. There was no difference in algal abundance between control plots and plots that received *D. antillarum* at any timepoint.

**Fig 4:**
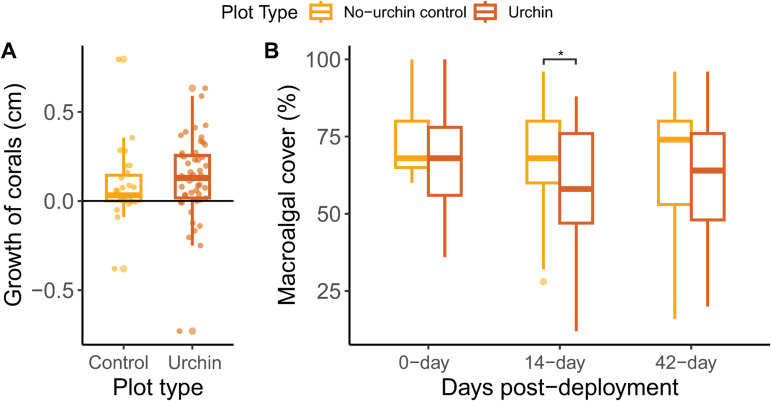
*Echniometra viridis* grazing. **(A)** Average growth of *Porites porites* corals (change in diameter, cm) over the 42-day experiment within control (*n *= 27 corals) and urchin plots (*n *= 49 corals) (Kruskal-Wallis, *χ*^2^ = 2.74, df = 1, p* *= 0.098). **(B)** Percent cover of macroalgae in the control plots (*n* = 2) and plots that received urchins (*n *= 4) over three monitoring time points for *Echinometra viridis*. Asterisk indicates significant differences between plots with and without urchins (GLMM, p <* *0.05).

The *E. viridis* deployment showed similar results, with higher retention in the high-complexity plots compared to the low-complexity plots across time (GLM, p* *< 0.05; Fig 3). After day 1, *E. viridis* had 80% retention in the high-complexity plots and 73% retention in the low-complexity plots for an overall site retention of 77%, and an additional 6% were found outside of the plots. By day 42, there was 33% retention in the high-complexity and 10% retention in the low-complexity plots (Fig 3). This species did not show an affinity for structure type within plots (GLM, p > 0.05). Benthic communities at control plots and plots that received urchins were similar at time of deployment. However, by day 14 (0.4 urchins m^-2^), algal cover was reduced within urchin plots compared to control plots (p* *< 0.05). By day 42, when urchin retention had fallen to 0.2 urchins m^-2^, algal cover was not significantly different between urchin and control plots (p* *= 0.12). Moreover, there was no significant difference between the growth rate of corals deployed within the control and urchin deployment plots (Kruskal-Wallis, *χ*^2 ^= 2.74, df = 1, p* *= 0.098; Fig 4).

In contrast, *L. variegatus* showed no preference for plot complexity or refuge type (GLM, p* *> 0.05; Fig 3). At the day 7 monitoring timepoint, *L. variegatus* had 18% retention in the high-complexity plots and 49% retention in the low-complexity plots. By day 14, only 5% of urchins remained in the high-complexity plots and at day 42, no urchins were observed. Retention at day 7 was different from all other monitoring time points (day 14 and day 42, p* *< 0.05). Algal cover between control plots and those that received *L. variegatus* did not differ at any survey time point.

#### Caging and site acclimation.

Retention of *D. antillarum* was higher over the 28-day period for individuals that were caged for one month prior to release, compared to urchins that were released without caging (GLM, p < 0.05). One day after deployment, there was 68% retention of urchins that were caged prior to deployment compared to 55% retention of uncaged urchins. By day 28, there was 17% retention of caged urchins and 0% retention of urchins released without a caging acclimation period.

**Fig 5:**
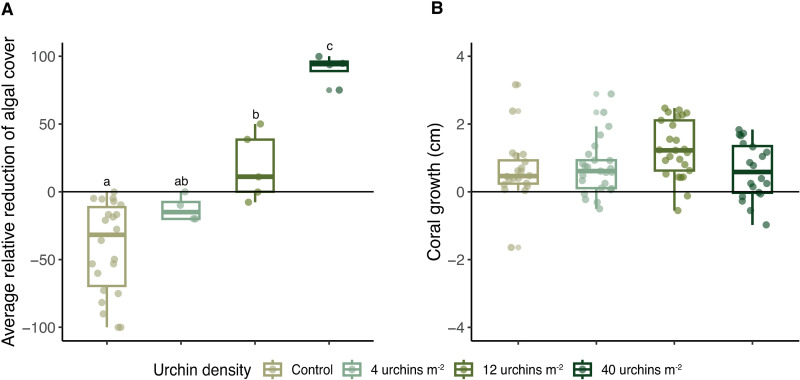
*Diadema antillarum* grazing based on density. **(A)** Relative reduction (%) of macroalgae cover in each urchin density treatment over the 28-day experiment. Each point indicates the relative reduction of algae in each cage, boxplots show the interquartile range for each urchin density, and letters denote groups with significant differences (F_3,31 _= 24.99, p < 0.001). **(B)** Growth (change in diameter, cm) of *Diploria labyrinthiformis* exposed to differing levels of grazing based on *Diadema antillarum* densities within cages after 28 days. Each point indicates a coral’s growth, boxplots show the interquartile range for each urchin density (LMM, p > 0.05).

**Fig 6:**
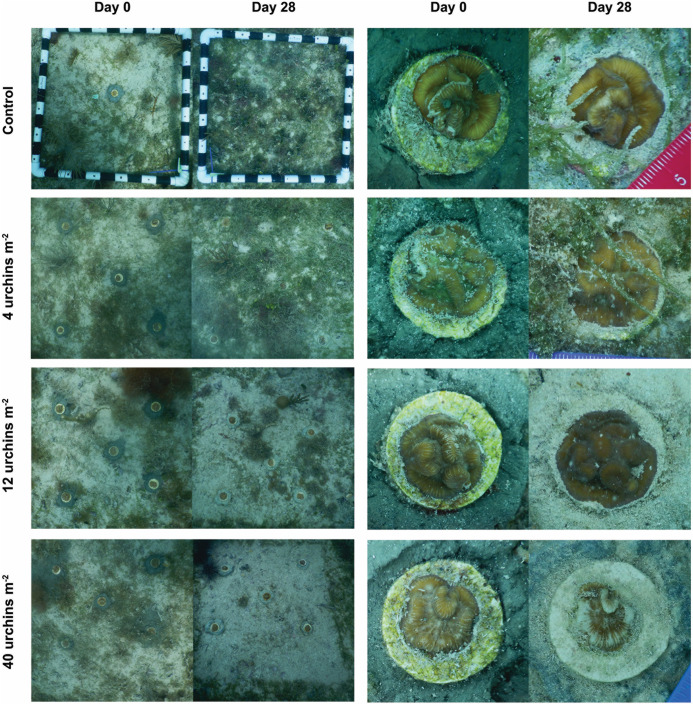
Impacts of urchin grazing on macroalgae and corals. Images of macroalgal cover exposed to different densities of caged *Diadema antillarum* (left) and *Diploria labyrinthiformis* fragments within the cages at deployment and 28 days post-caging (right).

Changes in algal cover were correlated with the density of caged urchins (Fig 5A). On average, the bottom was covered with 50–75% macroalgae at the beginning of the experiment. Seasonality influenced algal abundance at the site and there were increases in algal cover in both the control and 4 urchins m^-2^ treatments over the 28-day caging period, while there were decreases in the 12 urchins m^-2^ and 40 urchins m^-2^ treatments (Fig 5A). The control plots were different from the 12- and 40 urchins m^-2^ treatments, the 12- and 40 urchins m^-2^ treatments were different from each other, and the 4 urchins m^-2^ treatment was not significantly different from the control or 12 urchins m^-2^ density treatments (F_3,31 _= 24.99, p < 0.001; Fig 5A). Urchin density within cages also influenced the growth of small *D. labyrinthiformis* outplanted within cages. The corals within intermediate urchin densities (12 urchins m^-2^) grew, on average, more than the other three treatments; however no significant differences were detected (LMM, p > 0.05; Fig 5B). The corals in the control and the 4 urchins m^-2^ treatments were observed being overgrown by macroalgae such as *Caulerpa* and *Dictyota* (Fig 6). Corals in the 12 urchins m^-2^ treatment experienced neither contact algal competition nor signs of urchin grazing. In contrast, corals in the 40 urchins m^-2^ treatment averaged a 3.5% reduction in tissue cover caused by urchin overgrazing (Fig 6).

## Discussion

### Urchin retention within restoration plots

In an effort to increase the survivorship of outplanted corals and improve the efficiency of coral reef restoration, researchers are testing methods to enhance the conditions of the restoration plots and reef sites. One of the approaches being considered to enhance local conditions is to actively reduce macroalgal cover, which minimizes algal competition with coral recruits and outplants, by relocating sea urchins [17,44,59]. In this study, we evaluated the practicality and impacts of tandem coral-urchin restoration by deploying urchins of three species (wild *Echinometra viridis* and *Lytechinus variegatus,* and hatchery-reared *Diadema antillarum*) onto reef restoration plots in Miami, Florida, US. Our results highlighted low urchin retention and the potential of overgrazing when densities are too high as a major bottleneck in the use of urchins for algal control within restoration plots.

Urchins of the three species deployed (*D. antillarum*, *E. viridis*, and *L. variegatus*) left the plots relatively quick after deployment and did not display long-term retention within our site. Our efforts to enhance retention by adding natural (restored *A. cervicornis* colonies) and artificial (cement urchin “homes”) structure proved variable among survey times and species. *D. antillarum* had high retention within plots with large, complex *A. cervicornis* colony clusters (late restoration stage) only within the first 24 hours after deployment, while *E. viridis* had higher retention in complex plots up to six weeks after deployment. Moreover, the only individuals of *D. antillarum* found after day 1 were in high-complexity plots. This is in agreement with prior studies that showed that *D. antillarum* [64,65,71,72] and *E. viridis* prefer seeking shelter in complex and cryptic habitats [60,73,74].

As a consequence of “reef flattening” the quality and quantity of natural habitat and refuge has been reduced, thus the use of artificial structures has been suggested to increase urchin retention [18,33,50,65,75]. Within our restoration plots complexity influenced urchin retention, however, refuge type (live coral, dead coral, cement domes) did not. If high complexity can be achieved through coral outplanting, especially with large and complex branching *Acropora* colonies, there does not seem to be a need to enhance habitat complexity through the use of artificial structures. Outplanting of large, complex corals is not always an option so adding artificial structure could increase urchin retention within a site while coral cover and the refuge provided by outplants remain low. Ultimately, results of the present study suggest that what creates the shelter is not as important as the quality of the shelter itself. This outcome expands the portfolio of options for augmenting habitat complexity when stocking urchins into low-rugosity areas.

There are no previous studies on the restocking or translocation of *E. viridis* or *L. variegatus* in the Caribbean, so retention rates from this study cannot be compared to published rates. Surprisingly *E. viridis* had the highest retention of all three species and retention rates for *E. viridis* were higher than those previously reported for *D. antillarum*. This is likely a result of *E. viridis* having a small home and grazing range [60,76,77], making *E. viridis* a promising taxon for tandem restoration in the Caribbean. While *L. variegatus* has shown effective algal reduction in *ex-situ* systems, it’s grazing effect did not seem to translate to the reef environment. Another urchin taxon being considered for tandem reef restoration is *Tripneustes ventricosus,* but further research is needed on hatchery propagation methods and its application in tandem coral-urchin restoration [48,59].

Although low retention could also be attributed to predation, no visible signs (e.g., urchin spines or tests) were observed during *D. antillarum* or *E. viridis* deployments even when known predators and aggressors of the three urchin species were observed at the site (e.g., triggerfish, porcupine fish, snappers, grunts, damsel fish, and lobster) [78–80]. Direct predation was not observed, but the presence of these predator and aggressor species could influence the behavior and emigration rates of the urchins. Interestingly, signs of predation (spines and broken tests) were only observed during the *L. variegatus* deployment. This is not surprising as it is likely that *L. variegatus,* collected from seagrass beds, may not be suited for a reef environment and/or lack well-developed predator avoidance behavior [61,81,82]. Furthermore, the *L. variegatus* used in the study were larger than the other two species, which could have influenced their retention or predation susceptibility. The observation of spines and tests from the *L. variegatus* deployment suggest signs of predation for the other two species were likely not missed, supporting the hypothesis of urchin emigration.

While there have been previous *D. antillarum* restocking studies with higher retention values, these were achieved via caging (26 and 27% after 2 months) or placement in patch reefs surrounded by sand (26% and 20% after 17 months) to prevent emigration [18,46,48]. In a recent wild-relocation study completed at a restoration site in Miami, high retention of *D. antillarum* were observed (> 56% at 84 days and > 22% at 267 days after deployment) [44]. However, this study used wild-collected, adult urchins (49–105 mm test diameter, average TD = 72.65 mm) deployed in highly rugose habitat. Conversely, the present study utilized smaller, hatchery-reared *D. antillarum* that potentially exhibited reduced retention rates due to underdeveloped sheltering or predator avoidance behaviors, despite being reared with diurnal light cycles and tank structures to promote natural behavior [55,57]. A study conducted in Saba reported 25–30% retention of lab-reared and wild urchins after 10 days, which aligns with the results observed in this study [50]. The inherently mobile behavior of *D. antillarum* likely contributed to low retention, as individuals emigrated from areas with limited shelter in search of better refuge [64,65,83], often relocating more than 10 m away from the initial deployment plots.

Temporarily caging *D. antillarum* improved retention by allowing for acclimation without predation pressure, consistent with observations from previous studies in Puerto Rico (Williams, personal observations). However, caging is both time and resource-consuming, and may limit the number of urchins that can be deployed. Previous attempts at using large corrals to hold urchins in place were still met with emigration over time and loss/emigration of the urchins once barriers were removed [49]. However, while the urchins were held in place, they were able to significantly reduce algal abundance and increase coral survivorship and growth [48,66]. This also opens opportunities for artificially made reefs, where caging or sheltering structures could be integrated into the design to potentially increase long-term retention to control macroalgal cover. Caging urchins may only be practical in sites where urchins are still lost to emigration and predation even after an advanced restoration state has been achieved.

Emigration of urchins away from the desired restoration plots can be problematic for restoration efforts and may require the deployment of large numbers of urchins to achieve the desired outcome. However, fewer urchins may be needed to achieve a target density if the restoration site is highly complex, is not located within continuous reef habitat, and if urchins can be collected or grown to larger sizes. From a broader reef-scale perspective, urchins that emigrate continue to perform valuable ecological services, such as macroalgae grazing, despite relocation from intended target sites.

### Urchin grazing and coral impacts

The key goal of coral-urchin tandem restoration is to limit algal competition for outplanted corals at a time when they may be most vulnerable (e.g., small size and relocation stress) leading to an increase long-term coral survivorship. A similar approach is being evaluated for the tandem restoration of corals and the Caribbean king crab, *Maguimithrax spinosissimius*, which can significantly reduce algal cover and increase coral settlement at densities of 1 crab m^-2^ [20,84]. While field studies have shown that both wild and restocked *D. antillarum* can successfully decrease macroalgae abundances and enhance coral recruitment, the urchins need to persist at the site long enough and in sufficient densities to have a substantial impact [44,48,63,85–90].

A reduction in macroalgal cover was only observed in the present study during the *E. viridis* deployment, the species with the highest retention rates. The lack of grazing impacts for the other two species is likely related to their rapidly declining densities and low retention over time. This contrasts prior research where the benefits of herbivory by *D. antillarum* were seen at 0.15 urchins m^-2^ but no longer detectable at 0.04 urchins m^-2^ [44]. Nevertheless, a significant difference in macroalgal cover was observed at *E. viridis* densities of 0.4 individuals m^-2^ compared to plots that did not receive urchins. However, by the next survey, when *E. viridis* densities had fallen to 0.2 individuals m^-2^, differences in algal cover between plots were no longer observed. This suggests that the threshold for positive herbivory on Miami Beach reef falls between 0.2 and 0.4 urchins m^-2^. This is in contrast with studies from Panama where *E. viridis,* now filling the niche left by the die-off of *D. antillarum,* require densities of ≥ 15 urchins m^-2^ to have significant herbivory impacts [63,89]. The difference between these studies are likely attributable to variations in dominant algal communities and the behavior of *E. viridis*, which has a small grazing range and limited movement (< 19 cm a day) [60]. In our study, grazing impacts were measured in the area surrounding the refuge provided by restored corals and cement domes, thus showing algal reductions even at low urchin densities. While lower restocking densities may be needed for this species when refuge is provided, the benefits provided by these grazers may be limited to the vicinity of the restored corals. Another concern for the deployment of high densities of *E. viridis* in tandem restoration is the high levels of bioerosion exhibited by this excavator urchin [91]. Thus, a combination of grazers with different grazing ranges and behaviors will be needed to maximize herbivory benefits at scales beyond restoration plots.

The deployment of *D. antillarum* within cages during an acclimation period allowed us to evaluate the impacts of urchin density on algal cover and coral condition. As expected, impacts of grazing on algal cover were directly related to urchin density. While the largest reduction in algal cover may be desired to limit competition with corals, urchins have been shown to consume coral tissue and newly settled recruits when algal biomass is denuded [91–94]. The growth of small *D. labyrinthiformis* corals was highest within the cages with 12 urchins m^-2^ and was reduced at lower and higher urchin densities suggesting the presence of a grazing “sweet spot” that balances algal reduction and coral tissue removal. It is likely that at higher replications (and higher statistical power) significant differences between treatments may have been detected. This needs further exploration with additional experiments if coral-urchin tandem restoration is to be expanded. The need to curb algal biomass may decline as corals grow and thus reduce the density of urchins needed to control algae to limit bioerosion of the reef and loss of coral tissue [92,95,96]. The ideal density of *D. antillarum* still remains a topic of discussion. While some studies propose target densities of 1–3 urchins m^-2^ [17,18], others researchers found that a density of 1 urchin m^-2^ is insufficient to control algae on contemporary Caribbean reefs [88], consistent with our findings where 1 urchin m^-2^ had minimal impact on the algal community. Further research is needed to fully understand urchin retention and grazing dynamics on contemporary Caribbean coral reefs. Such knowledge will better inform restoration practitioners of the optimal urchin density within restoration plots and the frequency of restocking required to maintain those densities.

The benefits of tandem restoration will not be realized until urchin retention rates can be improved (recurrent deployments may be needed until urchins reach a sufficient or stable density). It has been suggested that there is a density dependence in *D. antillarum* recruitment, where the presence of adult urchins attracts early recruits and juveniles [97,98], while other studies show there is no density dependence [99] and have instead suggested that juvenile *D. antillarum* recruit to areas of grazed bare substrate [39]. Both in this study and in Puerto Rico, wild juvenile *D. antillarum* were spotted within the restocked adult plots, supporting the prediction that the presence of adults can enhance juvenile recruitment [48]. Once at a stable state, urchins can provide the necessary grazing needed to restore reef processes, such as an increase in coral cover, recruitment, and decrease in macroalgae [90,100,101]. Since retention rates of *D. antillarum* continue to be a bottleneck, it may be beneficial to aim for higher densities initially so that by the time the urchin population reaches a static density they will have the desired grazing effect.

## Conclusions

Based on the findings of this study, tandem coral-urchin restoration recommendations are as follows: 1) urchins need to be added to restored plots well before high coral mortality rates are observed (usually after 2 years for *A. cervicornis*), 2) restock urchins within areas of high reef complexity, either through the use of added shelter (live coral or artificial structures) or selecting sites with existing high rugosity, 3) restock urchins prior to coral spawning season to reduce macroalgal cover for coral larvae recruitment, 4) choose a restoration site that does not have a high abundance of predators (e.g., *Balistidae*, *Labridae*, and *Diodontidae*) to limit predation 5) use a combination of urchin species (e.g., *D. antillarum* and *E. viridis*) to maximize retention and herbivory, and 6) consider recurrent deployments of urchins or caging to maintain urchin densities initially, until a stable restoration state is met.

## Supporting information

S1 File Compiled data. (XLSX)
